# Blood Lead Concentrations and Depressive and Anxiety Symptoms in Childhood

**DOI:** 10.1001/jamanetworkopen.2025.56019

**Published:** 2026-01-28

**Authors:** Christian Hoover, George Papandonatos, Kim M. Cecil, Aimin Chen, Bruce P. Lanphear, Jeffrey R. Strawn, Kimberly Yolton, Joseph M. Braun

**Affiliations:** 1 Department of Epidemiology, Brown School of Public Health, Providence, Rhode Island; 2 Harvard Injury Control Research Center, Harvard T.H. Chan School of Public Health, Boston, Massachusetts; 3 Department of Biostatistics, Brown School of Public Health, Providence, Rhode Island; 4 Department of Pediatrics, Cincinnati Children’s Hospital Medical Center, University of Cincinnati College of Medicine, Cincinnati, Ohio; 5 Department of Environmental and Public Health Sciences, University of Cincinnati College of Medicine, University of Cincinnati, Cincinnati, Ohio; 6 Department of Radiology, University of Cincinnati College of Medicine, University of Cincinnati, Cincinnati, Ohio; 7 Department of Biostatistics, Epidemiology and Informatics, Perelman School of Medicine, University of Pennsylvania, Philadelphia; 8 Faculty of Health Sciences, Simon Fraser University, Burnaby, British Columbia, Canada; 9 BC Children’s Hospital Research Institute, Simon Fraser University, Burnaby, British Columbia, Canada; 10 Department of Psychiatry and Behavioral Neuroscience, Anxiety Disorders Research Program, College of Medicine, University of Cincinnati, Cincinnati, Ohio

## Abstract

**Importance:**

Low-level lead exposure during early brain development is associated with lower cognitive abilities and externalizing behavioral problems in children. However, the association of lead exposure with depression and anxiety remains understudied, particularly in later childhood, when these symptoms often manifest.

**Objective:**

To examine associations between low-level, serial blood lead concentrations in children and symptoms of depression and anxiety and to investigate for periods of susceptibility.

**Design, Setting, and Participants:**

This cohort study used data from a prospective population of 218 caregiver-child dyads (218 children and 218 parents in the Health Outcomes and Measures of Environment [HOME] Study) recruited from 2003 to 2006 in Cincinnati, Ohio. Children and their families were followed up from the second trimester to age 12 years (2016-2019). Data were analyzed between June 2024 to November 2025.

**Exposure:**

Serial blood lead concentrations in children were measured at ages 1, 2, 3, 4, 5, 8, and 12 years.

**Main Outcomes and Measures:**

We measured self- and caregiver-reported child depressive and anxiety symptoms at age 12 years using the Behavioral Assessment System for Children–3 (BASC-3), Children’s Depression Inventory–II (CDI-II), and Screen for Child Anxiety Related Disorders (SCARED).

**Results:**

Among 218 children (121 female [55.5%]; 78 Black [35.8%] and 140 White and other race or ethnicity [64.2%]; mean [SD] age, 12.4 [0.7] years), the median (IQR; range) mean within-child blood lead concentration was 9.6 (7.8 to 12.6; 4.8 to 32.4) μg/L. Each doubling in mean childhood blood lead concentrations was associated with increased risk of elevated child-reported depressive symptoms on the BASC-3 (relative risk [RR], 1.90; 95% CI, 1.00 to 3.66; *P* = .05) and increased risk of child- and caregiver-reported child depressive symptoms (RR, 1.76; 95% CI, 1.12 to 2.78; *P* = .02). By blood lead concentration, there were increasing adjusted mean differences in self-reported BASC-3 depression scores from age 1 year (1.82; 95% CI, −1.10 to 4.74; *P* = .22), a nonsignificant outcome, through age 8 years (3.22; 95% CI, 0.53 to 5.90; *P* = .02), a significant outcome. There were no associations between blood lead concentrations and self-reported depression measured with the Children’s Depression Inventory-II or anxiety measured with the SCARED. Child sex and race did not modify these associations.

**Conclusions and Relevance:**

In this study, low-level childhood blood lead concentrations were associated with self-reported depressive symptoms in later childhood, with particularly large increases in risk for exposures occurring in late childhood and early adolescence. These findings suggest that lead exposure during childhood may be associated with mental health in later childhood, highlighting the need for continued efforts to prevent lead exposure.

## Key Points

**Question:**

Is higher lead exposure during specific periods of childhood associated with increased depressive or anxiety symptoms in later childhood?

**Findings:**

In this cohort study of 218 caregiver-child dyads, increased childhood blood lead concentrations were associated with increased depressive symptoms in adolescence, with particularly large increases in depressive symptoms when exposure occurred later in childhood. No associations were observed with anxiety symptoms.

**Meaning:**

In this study, cumulative and later-childhood lead exposure was associated with increased risk of child depressive symptoms, emphasizing the need for continued efforts to reduce lead exposure throughout childhood.

## Teaser

This cohort study investigates the association of blood lead concentrations in specific childhood periods with depressive and anxiety symptoms in later childhood.

## Introduction

Lead is a neurotoxicant impacting cognitive and behavioral functioning.^1,2,3^ Young children are at risk for its harmful effects because of age-related behaviors (ie, hand-to-mouth contact) and are uniquely at risk because of their neurological development.^4^ Any exposure is associated with adverse health outcomes in children and adults.^5^ The Centers for Disease Control and Prevention (CDC) has established a reference value of 35 μg/L, which represents the 97.5th percentile of blood lead concentrations among US children in 2015 to 2018.^6^ Despite the reduced uses of lead, children are still exposed through legacy sources (eg, dust and soil).^7,8^

Lead exposure in children has been associated with social and behavioral problems but few studies have examined later psychiatric symptoms.^9,10,11^ In laboratory studies, developmental lead exposure (approximately 50 μg/L) in rat pups caused depressive symptoms, measured through forced swim tests.^12^ In another animal study,^13^ lead in drinking water induced persistent increases in anxiety-like behavior and inhibition of fear conditioning through reductions in neurogenesis and alteration of patterns of differentiation in newly born hippocampus cells. Human studies suggest that prenatal and infant lead exposures are associated with anxiety but not depression in childhood and adolescence, with outcomes dependent on exposure timing.^10,11,14^

Prospective studies have examined the association of lead exposure with internalizing symptoms across development. In a US cohort, Rokoff et al^10,11^ found that prenatal cord-blood lead concentrations (median, 11 μg/L), but not those taken serially between ages 1 and 3 years (median, 58 μg/L), were associated with elevated anxiety symptoms from childhood into early adulthood, particularly among girls. In contrast, blood lead concentrations in a New Zealand birth cohort at age 11 years (mean, 111 μg/L) were associated with increased internalizing symptoms and lifetime psychopathology in adulthood.^9^ Prior analyses^15^ from this cohort (the Health Outcomes and Measures of Environment [HOME] Study) reported associations between prenatal and early life exposures and externalizing behaviors, inattention, and executive functioning problems that extended into adolescence.

These findings underscore the long-term behavioral outcomes associated with early environmental exposures, provide context for our focus on internalizing symptoms during adolescence, and suggest that moderate prenatal and childhood lead exposures may be associated with emotional development. Few studies have evaluated serial blood-lead measures below 50 μg/L throughout childhood in association with child depression and anxiety. This is concerning given that these psychiatric conditions are prevalent among US adolescents.^16^ In 2022, 21% of adolescents reported elevated symptoms of anxiety and 17% reported symptoms of depression.^16^ Global estimates suggest that psychiatric disorders account for 1 in every 5 years of life lost due to disability, with undertreated mental illness associated with a more than 2-fold increase in the likelihood of early death.^17,18^ Given this burden and the ubiquity of lead, estimating associations of low-level lead exposure (≤50 μg/L) across the life span with symptoms of psychiatric illness becomes critical.^2,19^

To investigate whether lead exposures were associated with increased risk for depressive and anxious symptoms in children, we used a prospective cohort with serial blood lead measurements from ages 1 to 12 years and symptoms of depression and anxiety at age 12 years. We hypothesized that increased blood lead concentrations across childhood would be associated with increased depressive and anxiety symptoms in later childhood, with greater increases in risk for exposures occurring in later childhood.

## Methods

This cohort study is reported following the Strengthening the Reporting of Observational Studies in Epidemiology (STROBE) reporting guideline, and study protocols were approved by institutional review boards at the Cincinnati Children Hospital Medical Center and collaborating delivery hospitals (The Christ Hospital, University of Cincinnati Medical Center, and Good Samaritan Hospital). Data were analyzed between June 2024 to November 2025. All caregivers provided written informed consent for themselves and their children; children provided written informed assent at the age 12-year visit.

### Study Participants

We collected data from mother-child dyads recruited from 2003 to 2006 who were participating in the HOME Study in Cincinnati, Ohio, a prospective pregnancy and birth cohort described in detail elsewhere.^20,21^ Briefly, the cohort followed up 420 children from second trimester to early adolescence to assess associations of environmental toxicant exposures with various health outcomes. At approximately age 12 years (2016-2019), research staff assessed internalizing disorders (eg, anxiety and depression). Families resided in homes built before 1978 to capture children at increased risk for residential lead exposure.^20^

### Exposure: Blood Lead Concentrations

We collected whole venous blood at delivery (cord) and ages 1, 2, 3, 4, 5, 8, and 12 years. Blood samples were stored at −80 °C and transferred to CDC laboratories, where lead levels were quantified using inductively coupled plasma mass spectrometry methods.^22^ All batches included reagent blanks and quality control samples, with sample coefficient of variation less than 3.5%. Because not all children had blood lead measured at every visit and there was a linear decrease in blood lead with age, we used a regression calibration approach to impute missing lead concentrations and calculate mean blood lead concentrations at each visit while accounting for this linear trend. Among 339 children with at least 1 blood lead concentration measurement, we fit a linear mixed model with repeated log_2_-transformed blood lead concentrations as the outcome, a random intercept for participant, and a random slope for child age.^23^ Using participant-estimated best linear unbiased estimators from this model, we derived visit-specific blood lead concentrations at each age.

### Outcome: Mental Health Outcomes

We asked dyads to complete mental health questionnaires at a mean (range) child age of 12.4 (11-14) years, including the Behavioral Assessment System for Children-3 (BASC-3), a valid and reliable 160-item assessment that measures a broad range of behaviors, emotions, and adaptive skills.^24^ We focused on the Internalizing Composite score, which assess inwardly directed behaviors and emotions, including anxiety (excessive worry, fear, and nervousness), depression (sadness, hopelessness, and worthlessness), and somatization (physical complaints with no medical explanation, often linked to emotional distress). We converted raw scores to *T*-scores (mean [SD], 50 [10]). BASC-3 scores of 60 or greater reflect at-risk behaviors. In analyses of BASC-3 outcomes, we examined child, caregiver, and dyad (combined) scores. Adolescent self-report also included the Children’s Depression Inventory-II (CDI-II), a 28-item valid and reliable assessment of depressive symptoms in children.^25^ Scoring of the CDI-II included 6 subscales and a total score. For all CDI-II scores, raw values were converted to *T*-Scores with a mean of 50, where 65 or greater is considered clinically significant. For anxiety, we used the self-reported Screen for Child Anxiety Related Disorders (SCARED), a 41-item inventory evaluating symptoms across 5 subscales, with demonstrated validity and reliability in clinical and community settings.^26^ Clinically relevant symptoms are denoted by scores ranging from 3 or greater (Significant School Avoidance subscale) to 25 or greater (total score).

### Covariates

We selected covariates using a directed acyclic graph (eFigure 1 in Supplement 1) and include baseline and follow-up variables collected by trained study staff using standardized interviews and questionnaires, medical record (EHR) reviews, direct assessments, and environmental measures. Baseline variables included child sex assigned at birth (medical record; male vs female), mother’s education (parent report; ≤high school vs ≥some college), and race and ethnicity (parent report; categorized as non-Hispanic Black or White and other, which includes American Indian, Asian or Pacific Islander, Hispanic, White, and other). We assessed race and Hispanic ethnicity with 2 questions. We parameterized race as non-Hispanic Black, non-Hispanic White, and other races and ethnicities. We collapsed non-Hispanic White and the other category together because of the small sample size of the other category and because non-Hispanic Black individuals are at higher risk of lead exposure compared with members of other races and ethnicities.

Follow-up variables included child age, caregiver marital status, caregiver mental health, and caregiver-child relationship via the BASC-3 Parent Relationship Questionnaire, Relational Frustration subscale (BASC-3-PRQ).^27^ To assess caregiver mental health and avoid collinearity, we constructed a composite variable using principal component analysis of caregiver anxiety and depressive symptoms measured with Symptom Checklist-90-R anxiety and depressions subscales.^28^ These variables were strongly correlated (*r* = 0.57), and the first component explained 79% of variance in the 2 subscales.

### Statistical Analysis

We estimated univariate statistics of mean blood lead concentrations by covariates and associations of mean and age-specific concentrations with child psychiatric outcomes. This was done using multivariable linear regression to estimate mean differences in BASC-3, CDI-II, and SCARED scores per doubling in mean childhood blood lead concentration scaled to the SD of lead in the full sample (SD = 0.76). Because of the clustered nature of BASC-3 measurements, dyad reports were examined separately before combining and using generalized estimating equations (GEEs) to account for the correlation between reporters (child and caregiver).^29^ Second, we used modified Poisson regression with GEEs to estimate the risk ratio (RR) for at-risk BASC-3 and clinically significant CDI-II and SCARED scores per doubling in mean childhood blood lead concentrations. For combined scores, children were classified as having an elevated score if either caregiver or child report was 60 or greater.

Finally, we used a multiple informants model to examine associations of serial blood lead concentrations with BASC-3, CDI-II, and SCARED scores. For BASC-3 outcomes, we constructed separate models for child self-report only and caregiver reports. Briefly, the multiple informants model uses repeated blood lead concentrations to jointly estimate exposure-outcome associations at each age, determining whether associations differed across exposure periods.^30^ GEEs are used for parameter estimation that embed separate linear regression models for each period into a unified set of estimating equations.^23^ To assess for unique periods of risk, we considered interaction between blood lead concentration and exposure period, where *P* ≤ .15 was evidence of significant differences. All analyses used R statistical software version 4.5.1 (R Project for Statistical Computing) and significance noted for marginal (*P* ≤ .10) and statistically significant (*P* ≤ .05) outcomes; *P* values were 2-sided.^31^

For sensitivity, we calculated *E*-values to estimate the degree of residual confounding necessary to attenuate our results.^32^ We conducted sex- and race and ethnicity–stratified regressions to estimate strata-specific outcomes, while separate models with product interaction terms were used to assess heterogeneity of estimates. To examine potential overadjustment, we repeated analyses excluding caregiver mental health and BASC-PRQ. We also examined the association of childhood blood lead concentrations and BASC-3 hyperactivity for internal validity given lead’s association with externalizing behaviors. Additionally, we tested the lead-depression association considering a multiple informants model including cord blood lead levels to assess whether prenatal exposure meaningfully altered our results.^33,34^

## Results

### Univariate and Bivariate Associations

Of 420 children actively followed up in the cohort, 339 children had at least 1 blood lead concentration; 218 children and 218 parents had complete BASC-3 (combined 198 dyads) and 217 children had complete CDI and SCARED (described subsequently, in Table 1, and in eTable 1 in Supplement 1). The study sample consisted of 218 children (121 female [55.5%]; 78 Black [35.8%] and 140 White and other race or ethnicity [64.2%]; mean [SD] age, 12.4 [0.7] years). Caregiver mental health had the most missingness (18 caregivers [8.3%]), and missing values were multiply imputed by chained equations (20 imputations).^35,36^ In the full study sample, median blood lead concentrations declined from 12 to 144 months of age, with the greatest variability observed in early childhood (eFigure 5 in Supplement 1). Median concentrations were higher among males and Black children and those whose caregivers were unmarried or had less education; these concentrations displayed a monotonic increase with caregiver depression and decrease with child age (Table 1). The median (IQR; range) within-child mean blood lead concentration was 9.6 (7.8 to 12.6; 4.8 to 32.4) μg/L.

**Table 1.** Univariates Statistics of Childhood Blood Lead Concentrations

Variable	Participants, No. (%) (N = 218)	Blood lead concentration, median (IQR), μg/L	*P* value^a^
Child sex			
Female	121 (55.5)	9.1 (7.7-12.7)	.29
Male	97 (44.3)	10.1 (8.6-13.2)	
Child age, y			
11	19 (8.72)	12.0 (8.1-15.7)	.42
12	113 (51.8)	9.7 (7.9-12.0)	
13	71 (32.6)	9.0 (7.6-11.2)	
14	15 (6.9)	10.5 (8.5-15.2)	
Mother’s education			
≥College	167 (76.3)	8.9 (7.6-10.9)	<.001
≤High school	51 (23.3)	14.3 (11.8-19.0)	
Mother’s marital status			
Married	146 (67.0)	8.9 (7.5-10.7)	<.001
Not married	72 (33.0)	13.0 (9.0-16.6)	
Child race and ethnicity^b^			
Black	78 (35.78)	13.3 (9.9-16.4)	<.001
White and other	140 (64.2)	8.9 (7.5-10.5)	
**House lead dust loadings μg/ft^2^, quartile (range)**			
1 (0.0-0.6)	54 (24.8)	8.7 (7.5-11.1)	NA
2 (0.7-1.8)	54 (24.8)	9.4 (7.7-11.6)	
3 (1.9-5.70)	54 (24.8)	9.9 (8.7-14.0)	
4 (5.8-245.0)	54 (24.8)	11.4 (8.7-17.7)	
Missing	2 (1.0)	9.7 (9.0-10.5)	
**Caregiver anxiety, SCL-90R, quartile (range)**			
1 (37.0-37.0)	51 (23.4)	8.7 (7.4-10.5)	NA
2 (46.0-48.0)	50 (22.9)	9.6 (7.5-12.8)	
3 (44.0-52.0)	50 (22.9)	10.7 (8.2-15.1)	
4 (52.0-72.0)	50 (22.9)	10.0 (8.7-12.2)	
Missing	17 (7.8)	8.7 (7.7-9.8)	
**Caregiver depression, SCL-90R, quartile (range)**			
1 (34.0-46.0)	51 (23.4)	8.7 (7.4-10.5)	NA
2 (46.0-48.0)	50 (22.9)	9.6 (7.5-12.8)	
3 (48.0-57.0)	50 (22.9)	10.0 (8.8-13.3)	
4 (57.0-71.0)	50 (22.9)	11.1 (8.9-15.1)	
Missing	17 (7.8)	8.7 (7.7-9.8)	
**Relational frustration (BASC-PRQ), quartile^c^**			
1 (19.0-45.0)	55 (25.2)	12.2 (9.6-15.6)	NA
2 (45.0-52.0)	55 (25.2)	9.7 (8.1-11.2)	
3 (52.0-60.0)	54 (24.8)	8.8 (7.6-11.1)	
4 (60.0-63.0)	54 (24.8)	8.9 (7.4-10.4)	

Abbreviations: BASC-PRQ, Behavioral Assessment System for Children Parent Relationship Questionnaire, Relational Frustration subscale; NA, not applicable; SCL-90R, Symptom Checklist-90-R.

^a^ *P*-values derived from Kruskal-Wallis tests comparing median blood lead concentrations across groups.

^b^ Race and ethnicity were parent-reported. White and other includes American Indian, Asian or Pacific Islander, Hispanic, White non-Hispanic, and other.

^c^ Lower scores are reflective of worse parental relationships; therefore, quartile effects are expected to be in reverse.

### Depressive Symptoms (BASC-3 and CDI-II)

Mean lead concentrations were positively associated with child-reported depressive symptoms on the BASC-3. Associations had lower adjusted differences on the CDI-II. After adjustment, each SD increase in mean blood lead concentrations had a marginal 2.67-point (95% CI, −0.27 to 5.61; *P* = .08) higher mean of adolescence-reported BASC-3 depressive symptom score and no association for CDI-II (adjusted difference, 1.16; 95% CI, −0.89 to 3.21; *P* = .27) (eTables 2 and 3 and eFigures 2 and 3 in Supplement 1). Each doubling in lead was associated with an increase in the risk of elevated child-reported BASC-3 depression scores (RR, 1.90; 95% CI, 1.00-3.66; *P* = .05) and increased risk of child- and caregiver-reported child depressive symptoms (RR, 1.76; 95% CI, 1.12 to 2.78; *P* = .02) (Table 2). There was no association for caregiver-reported symptoms (RR, 1.49; 95% CI, 0.77-2.89; *P* = .24). We observed similar, marginal increases for subscales of the CDI-II, with an imprecise, marginal increase in risk of functional problems (RR, 1.94; 95% CI, 0.90-4.18; *P* = .09) (Table 3).

**Table 2.** Childhood Blood Lead Concentrations and Adjusted RR for At-Risk BASC-3 Scores

Variable	Participants, No. (N = 218)			Log-transformed blood lead concentration, median (IQR), μg/L^a^^,^^b^			At-risk BASC-3 scores, RR (95% CI)^b^^,^^c^^,^^d^					
	Child	Caregiver	Combined	Child	Caregiver	Combined	Child	*P* value	Caregiver	*P* value	Combined	*P* value
Internalizing problems composite												
Mean or below (<60)	174	198	372	9.1 (6.8-13.3)	9.4 (7.0-14.0)	9.2 (6.9-13.4)	NA	NA	NA	NA	NA	NA
At risk or above (≥60)	44	20	64	10.1 (8.1-17.8)	11.1 (7.0-17.7)	11.1 (7.6-17.8)	1.23 (0.67-2.27)	.51	2.67 (0.95-7.46)	.06	1.56 (0.91-2.68)	.11
**Anxiety**												
Mean or below (<60)	175	187	362	9.2 (7.0-13.4)	9.2 (6.8-14.2)	9.2 (6.9-13.5)	NA	NA	NA	NA	NA	NA
At risk or above (≥60)	43	31	74	10.9 (7.6-17.2)	10.0 (8.0-15.1)	10.3 (7.7-16.7)	1.14 (0.59-2.21)	.70	2.09 (0.96-4.55)	.06	1.44 (0.87-2.39)	.16
**Depression**												
Mean or below (<60)	184	189	373	9.2 (6.9-13.4)	9.2 (6.9-14.2)	9.2 (6.9-13.4)	NA	NA	NA	NA	NA	NA
At risk or above (≥60)	34	29	63	11.5 (8.5-18.4)	11.3 (7.5-17.7)	11.4 (7.9-17.9)	1.90 (1.00-3.66)	.05	1.49 (0.77-2.89)	.24	1.76 (1.12-2.78)	.02
**Somatization**												
Mean or below (<60)	190	195	385	9.6 (7.1-14.2)	9.4 (7.1-14.6)	9.5 (7.1-14.6)	NA	NA	NA	NA	NA	NA
At risk or above (≥60)	28	23	51	8.7 (6.8-13.3)	8.6 (6.8-11.9)	8.6 (6.8-12.3)	0.82 (0.30-2.23)	.70	1.28 (0.45-3.69)	.65	1.02 (0.49-2.39)	.96

Abbreviations: BASC-3, Behavioral Assessment System for Children–3; NA, not applicable; RR, risk ratio.

^a^ Childhood blood lead concentration was summarized for each child as the median of all available blood lead measurements from ages 1 to 12 years; values shown are the median (IQR) of this child-level measure within each symptom category.

^b^ Caregiver and child self-report results derived from modified Poisson regression models with robust standard errors. Combined results derived from linear regression models with GEE (compound symmetric covariance matrix) to account for the correlation of repeated measures within participants (2 measurements/participant), ensuring robust standard errors and valid inference despite the potential nonindependence of observations.

^c^ Adjusted for a principal component for caregiver anxiety and depression, maternal education (≤high school graduate or ≥college graduate), marital status (married or unmarried), child age (continuous in years), child sex (male or female), caregiver relationship (continuous), and race and ethnicity (non-Hispanic Black and White and other, including American Indian, Asian or Pacific Islander, Hispanic, White non-Hispanic, and other).

^d^ RRs are given per doubling in childhood blood lead concentration.

**Table 3.** Childhood Blood Lead Concentrations and Adjusted RR for Clinically Significant Scores

Variable	Participants, No. (%) (N = 217)	Blood lead concentration, median (IQR), μg/L^a^	Clinically significant score, RR (95% CI)^b^^,^^c^	*P* value
**CDI-II total score**				
Not clinically significant (<65)	198 (90.8)	9.2 (6.9-13.5)	NA	NA
Clinically significant (≥65)	20 (9.2)	11.2 (8.4-18.3)	1.19 (0.47-2.98)	.71
**CDI: emotional problems**				
Not clinically significant (<65)	198 (90.8)	9.4 (6.9-13.4)	NA	NA
Clinically significant (≥65)	20 (9.2)	10.4 (7.9-18.3)	1.43 (0.60-3.41)	.42
**CDI: negative mood**				
Not clinically significant (<65)	182 (83.5)	9.1 (6.8-13.0)	NA	NA
Clinically significant (≥65)	36 (16.5)	11.2 (8.3-18.7)	1.71 (0.87-3.35)	.12
**CDI: negative self-esteem**				
Not clinically significant (<65)	210 (96.3)	9.4 (7.0-13.5)	NA	NA
Clinically significant (≥65)	8 (3.7)	17.6 (7.1-21.2)	4.97 (1.12-22.02)	.04
**CDI: functional problems**				
Not clinically significant (<65)	194 (89.0)	9.0 (6.8-13.4)	NA	NA
Clinically significant (≥65)	24 (11.0)	11.5 (9.4-18.3)	1.94 (0.90-4.18)	.09
**CDI: interpersonal problems**				
Not clinically significant (<65)	202 (92.7)	9.4 (6.9-13.5)	NA	NA
Clinically significant (≥65)	16 (7.3)	11.4 (8.4-19.1)	1.30 (0.51-3.35)	.58
**CDI: ineffectiveness**				
Not clinically significant (<65)	183 (83.9)	8.9 (6.8-14.2)	NA	NA
Clinically significant (≥65)	35 (16.1)	11.3 (9.4-17.3)	1.27 (0.63-2.56)	.50
**SCARED total**				
Not anxious (<25)	142 (65.1)	8.9 (6.8-13.2)	NA	NA
Anxious (≥25)	76 (34.9)	10.3 (8.0-17.0)	1.10 (0.73-1.67)	.65
**SCARED: panic disorder or significant somatic symptoms**				
Not present (<7)	157 (72.0)	9.0 (6.9-14.2)	NA	NA
Present (≥7)	61 (28.0)	10.0 (8.1-14.6)	0.77 (0.44-1.37)	.38
**SCARED: generalized anxiety disorder**				
Not present (<9)	179 (82.1)	9.2 (6.9-14.6)	NA	NA
Present (≥9)	39 (17.9)	9.9 (7.7-12.8)	1.24 (0.60-2.53)	.56
**SCARED: separation anxiety disorder**				
Not present (<5)	137 (62.8)	8.9 (6.8-12.1)	NA	NA
Present (≥5)	81 (37.2)	10.3 (7.6-17.6)	0.96 (0.65-1.40)	.83
**SCARED: social anxiety disorder**				
Not present (<8)	162 (74.3)	9.4 (7.0-14.2)	NA	NA
Present (≥8)	56 (25.7)	9.7 (7.0-14.9)	0.87 (0.49-1.54)	.64
**SCARED: significant school avoidance**				
Not present (<3)	157 (72.0)	9.0 (6.9-12.2)	NA	NA
Present (≥3)	61 (28.0)	10.9 (8.0-18.1)	1.64 (1.07-2.50)	.02

Abbreviations: CDI, Children’s Depression Inventory; CDI-II, Children’s Depression Inventory–II; NA, not applicable; RR, risk ratio; SCARED, Screen for Child Anxiety Related Disorders.

^a^ Childhood blood lead concentration was summarized for each child as the median of all available blood lead measurements from ages 1 to 12 years; values shown are the median (IQR) of this child-level measure within each symptom category.

^b^ Adjusted for a principal component for caregiver anxiety and depression, maternal education (≤high school graduate or ≥college graduate), marital status (married or unmarried), child age (continuous, in years), child sex (male or female), caregiver relationship (continuous), and race and ethnicity (non-Hispanic Black or White and other, including American Indian, Asian or Pacific Islander, Hispanic, White non-Hispanic, and other). Results are derived from modified Poisson regression models with robust standard errors to account for potential nonnormality in residuals.

^c^ RRs are given per doubling in childhood blood lead concentration.

When examining periods of heightened susceptibility, increases in blood lead concentrations had adjusted differences in BASC-3 depression scores that grew from not significant at age 1 year (child adjusted difference, 1.82; 95% CI, −1.10 to 4.74; *P* = .22; caregiver adjusted difference, 1.39; 95% CI, −0.30 to 3.07; *P* = .11) to an association at 8 years for children (adjusted difference, 3.22; 95% CI, 0.53 to 5.90; *P* = .02) and a marginal outcome for caregivers at 8 years (adjusted difference, 1.85; 95% CI, −0.16 to 3.84; *P* = .07) before leveling somewhat by age 12 years, with an association for children (adjusted difference, 2.21; 95% CI, 0.19-4.24; *P* = .03) and a marginal outcome for caregivers (adjusted difference, 1.52; 95% CI, −0.24 to 3.29; *P* = .09) (Table 4). Associations of depressive and anxiety symptoms with blood lead concentrations did not vary by age of lead measurement (Table 4). The pattern of CDI-II scores by blood lead concentration was similar to that for BASC-3 depression scores, although outcomes were not significant (Table 4). There were no associations of lead concentrations with BASC-3 Internalizing Composite or Somatization scores (eTable 2 and eFigure 2 in Supplement 1).

**Table 4.** Adjusted Difference in Self- and Caregiver-Reported BASC-3 Depression and Anxiety by Age

Age, y	Child		Caregiver	
	Adjusted β (95% CI) (N = 218)^a^^,^^b^	*P* value	Adjusted β (95% CI) (N = 218)^a^^,^^b^	*P* value
**BASC-3: depression**				
1	1.82 (−1.10 to 4.74)	.22	1.39 (−0.30 to 3.07)	.11
2	2.07 (−0.87 to 5.00)	.17	1.50 (−0.27 to 3.27)	.10
3	2.31 (−0.62 to 5.24)	.12	1.60 (−0.24 to 3.45)	.09
4	2.55 (−0.35 to 5.46)	.09	1.70 (−0.21 to 3.61)	.08
5	2.78 (−0.08 to 5.64)	.06	1.78 (−0.18 to 3.74)	.08
8	3.22 (0.53 to 5.90)	.02	1.85 (−0.16 to 3.84)	.07
12	2.21 (0.19 to 4.24)	.03	1.52 (−0.24 to 3.29)	.09
Age × lead interaction	NA	.33	NA	.75
**BASC-3: anxiety**				
1	1.03 (−1.29 to 3.35)	.39	0.68 (−1.01 to 2.38)	.43
2	1.09 (−1.32 to 3.50)	.37	0.68 (−1.10 to 2.46)	.45
3	1.15 (−1.33 to 3.63)	.35	0.67 (−1.18 to 2.52)	.47
4	1.20 (−1.35 to 3.74)	.33	0.65 (−1.27 to 2.57)	.50
5	1.23 (−1.35 to 3.81)	.32	0.61 (−1.36 to 2.58)	.53
8	1.21 (−1.34 to 3.76)	.34	0.44 (−1.56 to 2.45)	.66
12	0.77 (−1.27 to 2.81)	.46	0.12 (−1.60 to 1.85)	.91
Age × lead interaction	NA	.99	NA	.97
**CDI-II total**				
1	0.78 (−1.10 to 2.66)	.41	NA	NA
2	0.88 (−1.05 to 2.81)	.37	NA	NA
3	0.99 (−0.98 to 2.95)	.32	NA	NA
4	1.09 (−0.90 to 3.08)	.28	NA	NA
5	1.18 (−0.81 to 3.17)	.24	NA	NA
8	1.37 (−0.53 to 3.26)	.16	NA	NA
12	1.05 (−0.51 to 2.60)	.19	NA	NA
Age × lead interaction	NA	.85	NA	NA
**SCARED total**				
1	−0.42 (−3.13 to 2.29)	.76	NA	NA
2	−0.35 (−3.17 to 2.48)	.81	NA	NA
3	−0.26 (−3.18 to 2.67)	.86	NA	NA
4	−0.15 (−3.16 to 2.86)	.92	NA	NA
5	−0.04 (−3.11 to 3.04)	.98	NA	NA
8	0.33 (−2.76 to 3.43)	.83	NA	NA
12	−0.13 (−2.64 to 2.39)	.92	NA	NA
Age × lead interaction	NA	.75	NA	NA

Abbreviations: BASC-3, Behavioral Assessment System for Children–3; CDI-II, Children’s Depression Inventory–II; NA, not applicable; SCARED, Screen for Child Anxiety Related Disorders.

^a^ Adjusted for a principal component for caregiver anxiety and depression, maternal education (≤high school graduate or ≥college graduate), marital status (married or unmarried), child age (continuous in years), child sex (male or female), caregiver relationship (continuous), and race and ethnicity (non-Hispanic Black and White and other, including American Indian, Asian or Pacific Islander, Hispanic, White non-Hispanic, and other).

^b^ Differences are given per SD increase in log_2_-transformed childhood blood lead concentrations and rescaled by the SD of the full sample.

### Anxiety Symptoms: BASC-3 and SCARED

Generally, children’s blood lead concentrations were not associated with BASC-3 anxiety or SCARED scores (Table 4; eTables 2 and 3 and eFigures 2 and 4 in Supplement 1). However, there was a marginal outcome for lead and BASC-3 anxiety scores for caregiver report (RR, 2.09; 95% CI, 0.96-4.55; *P* = .06) and no association for child report (RR, 1.14; 95% CI, 0.59-2.21; *P* = .70) (Table 2). Results on the SCARED were null, although there was significantly higher risk of SCARED Significant School Avoidance (RR, 1.64; 95% CI, 1.07-2.50; *P* = .02) per doubling in mean blood lead concentrations, also mirrored in analysis of continuous scores (eTable 3 in Supplement 1). There were no significant differences by serial blood lead concentration with BASC-3 anxiety or SCARED (Table 4).

### Sensitivity and Stratified Interaction Association Analysis

Blood lead concentrations and child-reported BASC-3 depression and anxiety, CDI-II, and SCARED outcomes were not significantly modified by child sex or race and ethnicity (eTable 4 in Supplement 1). For the association between concentrations and risk of elevated BASC-3 child-reported depression scores, a confounder would have to be associated with both lead and depression with an RR of at least 3.21 (*e*-value*)* to attenuate the observed association to null. Childhood lead concentration had associations with hyperactivity outcomes assessed via child report (adjusted difference, 3.01; 95% CI, 0.32-5.69; *P* = .03) and caregiver report (adjusted difference, 2.48; 95% CI, 0.34-4.64; *P* = .02).

When excluding caregiver mental health and BASC-PRQ, blood lead concentrations had depressive symptom outcomes that increased in magnitude and precision but remained consistent in pattern (Table 5). Anxiety outcomes on the BASC-3 and SCARED remained small and nonsignificant, and there were no significant age × lead differences. Cord blood lead was associated with depressive symptoms in later childhood (eTable 5 in Supplement 1).

**Table 5.** Adjusted Difference in BASC-3, CDI-II, and SCARED Outcomes by Childhood Blood Lead Concentrations Omitting BASC-PRQ and Principal Component of Parent Depression and Anxiety

Age, y	Child		Caregiver	
	Adjusted β (95% CI) (N = 218)^a^^,^^b^	*P* value	Adjusted β (95% CI) (N = 218)^a^^,^^b^	*P* value
**BASC-3: depression**				
1	2.39 (−0.61 to 5.39)	.12	2.06 (0.30 to 3.82)	.02
2	2.65 (−0.36 to 5.67)	.09	2.20 (0.35 to 4.06)	.02
3	2.91 (−0.10 to 5.93)	.06	2.33 (0.40 to 4.27)	.02
4	3.16 (0.17 to 6.15)	.04	2.44 (0.43 to 4.46)	.02
5	3.38 (0.43 to 6.34)	.03	2.53 (0.45 to 4.60)	.02
8	3.76 (0.96 to 6.57)	.008	2.55 (0.42 to 4.68)	.02
12	2.74 (0.59 to 4.88)	.01	2.20 (0.33 to 4.07)	.02
Age × lead interaction	NA	.26	NA	.99
**BASC-3: anxiety**				
1	1.25 (−0.98 to 2.49)	.27	1.02 (−0.78 to 2.82)	.27
2	1.33 (−0.99 to 3.65)	.26	1.05 (−0.84 to 2.93)	.28
3	1.39 (−1.00 to 3.78)	.26	1.07 (−0.90 to 3.03)	.29
4	1.44 (−1.00 to 3.89)	.25	1.07 (−0.96 to 3.10)	.30
5	1.48 (−1.01 to 3.96)	.25	1.06 (−1.03 to 3.14)	.32
8	1.44 (−1.02 to 3.90)	.25	0.91 (−1.20 to 3.03)	.40
12	1.00 (−1.00 to 2.98)	.33	0.58 (−1.23 to 2.39)	.53
Age × lead interaction	NA	.97	NA	.96
**CDI-II total**				
1	1.42 (−0.42 to 3.26)	.13	NA	NA
2	1.54 (−0.35 to 3.43)	.11	NA	NA
3	1.66 (−0.27 to 3.59)	.09	NA	NA
4	1.77 (−0.20 to 3.73)	.08	NA	NA
5	1.86 (−0.12 to 3.83)	.07	NA	NA
8	1.96 (0.04 to 3.89)	.05	NA	NA
12	1.62 (0.03 to 3.21)	.05	NA	NA
Age × lead interaction	NA	.62	NA	NA
**SCARED total**				
1	0.02 (−2.55 to 2.60)	.99	NA	NA
2	0.12 (−2.56 to 2.80)	.93	NA	NA
3	0.23 (−2.54 to 3.00)	.87	NA	NA
4	0.34 (−2.50 to 3.19)	.82	NA	NA
5	0.47 (−2.44 to 3.37)	.76	NA	NA
8	0.81 (−2.13 to 3.76)	.59	NA	NA
12	0.34 (−2.07 to 2.74)	.78	NA	NA
Age × lead interaction	NA	.87	NA	NA

Abbreviations: BASC-3, Behavioral Assessment System for Children–3; BASC-PRQ, Behavioral Assessment System for Children Parent Relationship Questionnaire, Relational Frustration subscale; CDI-II, Children’s Depression Inventory–II; NA, not applicable; SCARED, Screen for Child Anxiety Related Disorders.

^a^ Adjusted for a principal component analysis for caregiver anxiety and depression, maternal education (≤high school graduate or ≥college graduate), marital status (married or unmarried), child age (continuous in years), child sex (male or female), caregiver relationship (continuous), and race and ethnicity (non-Hispanic Black or White and other, including American Indian, Asian or Pacific Islander, Hispanic, White non-Hispanic, and other).

^b^ Differences are given per SD change in age-specific childhood blood lead concentrations.

## Discussion

In this prospective cohort study, higher childhood blood lead concentrations were associated with increased child depressive symptoms. Blood lead concentrations measured more proximal to adolescence appeared similar to prenatal exposures in strength and magnitude. We found few notable associations with child anxiety, except for the SCARED school avoidance subscale. Lending internal validity to our results and consistent with prior studies, mean blood lead concentrations were associated with elevated hyperactive scores.^33,34^

Prior research suggests that prenatal lead exposure is associated with later depressive symptoms.^10,11,37^ Lin and colleagues^37^ found that among 680 adults (mean age, 62 years), biomarkers for prenatal exposure (lead concentrations reconstructed from dentine layers of shed deciduous teeth) were associated with an increase in the odds of depression (odds ratio, 1.72; 95% CI, 1.20-2.99) among 31 adults compared with a lower odds ratio for postnatal exposure (1.60; 95% CI, 0.99-2.58). Separately, in a cohort of 468 children, prenatal cord lead concentrations were associated with elevated anxiety symptoms in early adulthood compared with postnatal exposures (peak value between 1-3 years), which were not associated with later internalizing symptoms.^10,11^ These studies share many similarities with ours; when cord blood was included in our study, it was associated with later depressive symptoms, although this did not change the strong and more precise association observed at age 8 years. Our findings are also consistent with prior work in the HOME Study,^15^ which identified age 8 years as a period of heightened susceptibility for lead’s association with externalizing behaviors.

Several biological mechanisms could explain how lead exposure may be associated with psychiatric illness. Animal studies suggest that lead may alter neurotransmitter function, reduce neurogenesis, and disrupt synaptic plasticity, particularly in brain regions associated with mood regulation.^12,13,38^ These changes can lead to behavior resembling internalizing disorders.^12,39^ Other proposed mechanisms include oxidative stress, inflammation, and epigenetic modifications that may contribute to the onset and persistence of mental health symptoms after lead exposure.^40,41,42^

Among children, anxiety symptoms may precede depression, and some studies suggest that anxiety masks caregiver appraisal of depressive symptoms.^19,43^ This dynamic could explain why child-reported depressive symptoms showed associations with greater increases in risk than caregiver-reported symptoms. Other studies suggest that anxiety and depressive symptoms overlap, complicating the ability to distinguish the 2 conditions in youth.^44^ Symptoms of these conditions may also fluctuate, thus necessitating longitudinal assessment. Indeed, prior literature largely focuses on the lead-internalizing association in childhood rather than adolescence and young adulthood, when symptoms are more likely to emerge.^44,45^

It was notable that there were associations with the BASC-3 but not CDI-II. The BASC-3 captures a broad range of emotional and behavioral features, including withdrawal, lack of interest in activities, and social detachment, in addition to mood-related symptoms, which have previously been associated with lead exposure.^46^ The CDI-II assesses internal cognitive and emotional symptoms, potentially missing some behavioral expressions of depression that may be associated with lead exposure.^25^ These scale-specific differences are informative, suggesting that lead may be preferentially associated with behavioral-affective rather than purely cognitive-emotional dimensions of depression. Similarly, the smaller RRs in associations and marginal outcomes observed for anxiety may indicate that lead’s neurotoxic outcomes are associated with greater outcomes in affective blunting and dysphoria than hyperarousal or worry.^12^ This pattern underscores the importance of assessing multiple domains of internalizing symptoms to capture the full spectrum of lead’s potential associated psychological outcomes. After removal of BASC-PRQ and parent mental illness, outcomes increased in magnitude and precision, which was consistent with these variables operating as intermediates rather than confounders.

### Limitations

This study has several limitations. First, self-reported measures of mental health are subject to recall and reporting bias, although validated self-report instruments in children show moderate to strong agreement with structured clinical interviews. The use of both self- and caregiver reports enhances the robustness of our findings by capturing child mental health in separate contexts, reducing the likelihood that symptoms were overlooked.^29,47,48^ Second, the sample is limited to a specific geographic region, potentially limiting the generalizability of our findings to other populations with different environmental or sociodemographic characteristics. However, the sociodemographic diversity of our sample provides a meaningful representation of urban populations in similar regions. Notably, the HOME Study cohort was recruited from families living in older housing to capture children at elevated environmental lead exposure risk, but median blood lead concentrations were comparable to those observed nationally during the same period.^20^ Third, we imputed some blood lead concentrations, which makes assumptions about the nature of missingness. However, if these assumptions were met, then our modeled blood lead concentrations would more accurately represent concentrations given that they accounted for the linear decrease with age. Moreover, our study has several innovations compared with other studies because we had multiple serial blood lead measures later in childhood, with prospective assessments of child internalizing symptoms.^9,49,50,51,52^

## Conclusions

This cohort study found that childhood lead exposure was associated with more depressive symptoms in later childhood, particularly when measured at delivery and age 8 years. These findings suggest that later childhood lead exposures may be equally or more neurotoxic than early childhood lead exposure. Future studies should continue to explore how to prevent cumulative or later childhood lead exposure, as well as whether there exist distinct longitudinal patterns of lead exposure that may be associated with child mental health outcomes.

## Notes

Supplement 1. eTable 1. Univariates statistics of child and caregiver reported BASC-3 Depression and Anxiety, CDI-II and SCARED total scores eTable 2. Adjusted difference in child, caregiver, and combined (cluster size of 2) BASC-3 outcomes per SD increase in log_2_-transformed mean childhood blood lead concentrations (rescaled by SD of full sample) eTable 3. Adjusted difference in CDI-II (depression) and SCARED (anxiety) scores per SD change in the log_2_-transformed mean childhood blood lead concentrations (rescaled by SD of full study sample) eTable 4. Stratified and interaction effect models examining the adjusted differences in the log_2_-transformed blood lead concentrations and BASC-3: Depression and Anxiety (child self-report) and CDI-II and SCARED child outcomes (rescaled to SD of full sample) eTable 5. Adjusted difference in BASC-3, CDI-II, and SCARED outcomes per SD change in neonatal cord and age-specific log_2_-transformed childhood blood lead concentrations (rescaled by SD of full sample) eFigure 1. Directed acyclic graph of exposures, outcomes, and covariates eFigure 2. Adjusted difference in child, caregiver, and combined (cluster size of 2) BASC-3 outcomes per SD increase in log_2_-transformed mean childhood blood lead concentrations (rescaled by SD of full sample) eFigure 3. Unadjusted and adjusted difference in CDI-II (depression) scores per SD change in the log_2_-transformed mean childhood blood lead concentrations (n = 217) (rescaled by SD of full study sample) eFigure 4. Unadjusted and adjusted differences in SCARED (anxiety) scores per SD change in the log-transformed mean childhood blood lead concentrations (rescaled by SD of full study sample) eFigure 5. Serial whole blood lead concentrations (µg/L) across childhood (n = 339)

Supplement 2.
Data Sharing Statement
