# Supplementary material for: Blood Lead Concentrations and Depressive and Anxiety Symptoms in Childhood
Source: JAMA Netw Open. 2026 Jan 28;9(1):e2556019. doi: 10.1001/jamanetworkopen.2025.56019 (PMC12853203; doi:10.1001/jamanetworkopen.2025.56019)
Supplement: Supplement 1. — eTable 1. Univariates statistics of child and caregiver reported BASC-3 Depression and Anxiety, CDI-II and SCARED total scores eTable 2. Adjusted difference in child, caregiver, and combined (cluster size of 2) BASC-3 outcomes per SD increase in log2-transformed mean childhood blood lead concentrations (rescaled by SD of full sample) eTable 3. Adjusted difference in CDI-II (depression) and SCARED (anxiety) scores per SD change in the log2-transformed mean childhood blood lead concentrations (rescaled by SD of full study sample) eTable 4. Stratified and interaction effect models examining the adjusted differences in the log2-transformed blood lead concentrations and BASC-3: Depression and Anxiety (child self-report) and CDI-II and SCARED child outcomes (rescaled to SD of full sample) eTable 5. Adjusted difference in BASC-3, CDI-II, and SCARED outcomes per SD change in neonatal cord and age-specific log2-transformed childhood blood lead concentrations (rescaled by SD of full sample) eFigure 1. Directed acyclic graph of exposures, outcomes, and covariates eFigure 2. Adjusted difference in child, caregiver, and combined (cluster size of 2) BASC-3 outcomes per SD increase in log2-transformed mean childhood blood lead concentrations (rescaled by SD of full sample) eFigure 3. Unadjusted and adjusted difference in CDI-II (depression) scores per SD change in the log2-transformed mean childhood blood lead concentrations (n = 217) (rescaled by SD of full study sample) eFigure 4. Unadjusted and adjusted differences in SCARED (anxiety) scores per SD change in the log-transformed mean childhood blood lead concentrations (rescaled by SD of full study sample) eFigure 5. Serial whole blood lead concentrations (µg/L) across childhood (n = 339) [file jamanetwopen-e2556019-s001.pdf]

Supplemental Online Content

Hoover C, Papandonatos G, Cecil KM, et al. Blood lead concentrations and depressive and anxiety symptoms in childhood. *JAMA Netw Open*. 2026;9(1):e2556019.
doi:10.1001/jamanetworkopen.2025.56019

eTable 1. Univariates statistics of child and caregiver reported BASC-3 Depression and Anxiety, CDI-II and SCARED total scores

eTable 2. Adjusted difference in child, caregiver, and combined (cluster size of 2) BASC-3 outcomes per SD increase in log_2-transformed mean childhood blood lead concentrations (rescaled by SD of full sample)

eTable 3. Adjusted difference in CDI-II (depression) and SCARED (anxiety) scores per SD change in the log_2-transformed mean childhood blood lead concentrations (rescaled by SD of full study sample)

eTable 4. Stratified and interaction effect models examining the adjusted differences in the log_2-transformed blood lead concentrations and BASC-3: Depression and Anxiety (child self-report) and CDI-II and SCARED child outcomes (rescaled to SD of full sample)

eTable 5. Adjusted difference in BASC-3, CDI-II, and SCARED outcomes per SD change in neonatal cord and age-specific log_2-transformed childhood blood lead concentrations (rescaled by SD of full sample)

eFigure 1. Directed acyclic graph of exposures, outcomes, and covariates

eFigure 2. Adjusted difference in child, caregiver, and combined (cluster size of 2) BASC-3 outcomes per SD increase in log_2-transformed mean childhood blood lead concentrations (rescaled by SD of full sample)

eFigure 3. Unadjusted and adjusted difference in CDI-II (depression) scores per SD change in the log_2-transformed mean childhood blood lead concentrations (n = 217) (rescaled by SD of full study sample)

eFigure 4. Unadjusted and adjusted differences in SCARED (anxiety) scores per SD change in the log-transformed mean childhood blood lead concentrations (rescaled by SD of full study sample)

eFigure 5. Serial whole blood lead concentrations (µg/L) across childhood (n = 339)

This supplemental material has been provided by the authors to give readers additional information about their work.

© 2026 Hoover C et al. *JAMA Network Open*.

eTable 1. Univariates statistics of child and caregiver reported BASC-3 Depression and Anxiety, CDI-II and SCARED total scores [a]: The HOME Study (n=218).[a]

Outcome Measure	Minimum	25th	50th	75th	Maximum	Mean	SD
BASC-3 Internalizing Composite: Adolescent Report	35.00	42.00	47.50	56.00	102.00	50.90	12.30
BASC-3 Internalizing Composite: Caregiver Report	35.00	44.00	48.00	54.00	80.00	49.20	8.25
BASC-3 Anxiety: Adolescent Report	33.00	41.00	48.00	56.00	83.00	50.00	11.20
BASC-3 Anxiety: Caregiver Report	33.00	43.20	48.00	54.00	80.00	49.50	8.90
BASC-3 Depression: Adolescent Report	40.00	42.00	45.00	54.00	108.00	50.40	13.50
BASC-3 Depression: Caregiver Report	38.00	44.00	48.00	55.00	88.00	50.20	8.95
BASC-3 Somatization: Adolescent Report	42.00	42.00	42.00	49.00	91.00	48.10	9.18
BASC-3 Somatization: Caregiver Report	37.00	42.00	47.00	53.00	81.00	48.40	8.10
CDI-II Total	40.00	44.00	49.00	56.00	90.00	51.50	10.00
SCARED Total	1.00	11.00	20.00	30.00	65.00	21.40	12.90

[a]Pearson correlations between child and caregiver BASC-3 scores were 0.48 (Depression), 0.30 (Anxiety), 0.38 (Internalizing Problems), and 0.37 (Somatization).

© 2026 Hoover C et al. *JAMA Network Open*.

eTable 2. Adjusted [a] difference in child, caregiver, and combined (cluster size of 2)[b] BASC-3 outcomes per standard deviation increase in log_2-transformed average childhood blood lead concentrations (rescaled by SD of full sample): The HOME Study (n=218).[a]

BASC-3 Outcomes	Child Adjusted ß (95% CL)	Caregiver Adjusted ß (95% CL)	Combined[b] Adjusted ß (95% CL)
Internalizing Problems[c]	2.03 (-0.65, 4.71)	0.57 (-1.38, 2.51)	1.53 (-0.20, 3.26)*
Anxiety	1.21 (-1.40, 3.82)	0.60 (-1.40, 2.61)	0.91 (-1.24, 2.29)
Depression	2.67 (-0.27, 5.61)*	1.80 (-0.22, 3.82)*	2.23 (0.38, 4.09)**
Somatization	0.07 (-2.25, 2.38)	0.25 (-1.94, 2.44)	0.16 (-1.44, 1.76)

[a] Adjusted for: A principal component analysis for caregiver anxiety and depression, maternal education (high school graduate or less, college graduate or above), marital status (married, unmarried), child age (continuous, years), child sex (male, female), caregiver relationship (continuous) and race (Non-Hispanic Black, White and Other; including White, non-Hispanic; Hispanic; American Indian; Asian/Pacific Islander; and Other).
[b] Combined results derived from linear regression models with GEE (compound symmetric covariance matrix) to account for the correlation of repeated measures within participants, ensuring robust standard errors and valid inference despite the non-independence of observations.
[c] Composite scale of anxiety, depression, and somatization.
*Marginally significant at p≤.10
**Statistically significant at p≤.05

© 2026 Hoover C et al. *JAMA Network Open*.

eTable 3. Adjusted[a] difference in CDI-II (depression) and SCARED (anxiety) scores per standard deviation change in the log_2-transformed average childhood blood lead concentrations (rescaled by standard deviation of full study sample): The HOME Study (n=218).

**CDI-II**	**Adjusted ß (95% CL)**
Total Score	1.16 (-0.89, 3.21)
Emotional Problems	1.38 (-0.66, 3.41)
Negative Mood	1.66 (-0.74, 4.05)
Negative Self-Esteem	0.63 (-1.25, 2.50)
Functional Problems	1.04 (-1.12, 3.41)
Interpersonal Problems	0.36 (-1.91, 2.64)
Ineffectiveness	1.25 (-1.01, 3.51)
**SCARED**	
Total Score	-0.18 (-3.30, 2.94)
Panic Disorder or Significant Somatic Symptoms	-0.17 (-1.24, 0.91)
Generalizing Anxiety Disorder	-0.01 (-0.91, 0.92)
Separation Anxiety Disorder	-0.12 (-0.84, 0.59)
Social Anxiety Disorder	-0.33 (-1.20, 0.54)
Significant School Avoidance	0.40 (0.04, 0.77)**

[a] Adjusted for: A principal component analysis for caregiver anxiety and depression, maternal education (high school graduate or less, college graduate or above), marital status (married, unmarried), household dust lead loadings at baseline (continuous, log10-transformed), child age (continuous, years), child sex (male, female), caregiver relationship (continuous) and race (Non-Hispanic Black, White and Other; including White, non-Hispanic; Hispanic; American Indian; Asian/Pacific Islander; and Other).
*Marginally significant at p≤.10
**Statistically significant at p≤.05

© 2026 Hoover C et al. *JAMA Network Open*.

eTable 4. Stratified and interaction effect models examining the adjusted differences in the log_2-transformed blood lead concentrations and BASC-3: Depression and Anxiety (child self-report) and CDI-II and SCARED child outcomes (rescaled to SD of full sample): The HOME Study (n=218).

	**BASC-3: Depression ß (95% CL)**	**BASC-3: Anxiety ß (95% CL)**	**CDI-II ß (95% CL)**	**SCARED ß (95% CL)**
Stratified Analysis				
Boys	3.78 (-0.19, 7.74)	2.50 (-0.77, 5.77)	1.78 (-0.55, 4.10)	0.11 (-3.49, 3.71)
Girls	1.26 (-2.54, 5.06)	-0.14 (-4.30, 4.03)	0.77 (-2.50, 4.04)	-0.77 (-5.80, 4.26)
NH Black	2.14 (-2.79, 7.08)	-0.30 (-4.45, 3.86)	1.39 (-2.12, 4.90)	-2.74 (-7.90, 2.41)
White & Other	2.85 (-0.43, 6.13)	2.29 (-0.92, 5.51)	0.80 (-1.56, 3.16)	1.59 (-2.33, 5.51)
Interaction Term				
Gender	0.59 (-3.62, 4.79)	1.83 (-2.40, 6.07)	-1.06 (-4.57, 2.46)	-1.34 (-6.31, 3.62)
Race	1.06 (-4.12, 6.24)	2.53 (-2.31, 7.38)	1.24 (-3.03, 5.50)	4.26 (-1.96, 10.48)

*Interaction product term for effect modification significance assessed at p≤.15

© 2026 Hoover C et al. *JAMA Network Open*.

eTable 5. Adjusted[a] difference in BASC-3, CDI-II and SCARED outcomes per standard deviation change in neonatal cord and age-specific log_2-transformed childhood blood lead concentrations (rescaled by SD of full sample): The HOME Study (n=218).

	Child	Caregiver
	Adjusted ß (95% CI)	
**BASC-3 - Depression**		
Birth (Cord)	4.85 (0.48, 9.22)**	2.41 (-1.02, 5.83)
1 Year	1.78 (-1.03, 4.58)	1.33 (0.30, 2.95)
2 Year	2.02 (-0.79, 4.82)	1.44 (0.35, 3.14)*
3 Year	2.26 (-0.54, 5.06)	1.55 (0.40, 3.32)*
4 Year	2.50 (-0.27, 5.27)*	1.64 (0.43, 3.47)*
5 Year	2.72 (-0.00, 5.45)*	1.71 (0.45, 3.60)*
8 Year	3.16 (0.59, 5.74)**	1.79 (0.42, 3.72)*
12 Year	2.18 (0.23, 4.12)**	1.48 (-0.25, 3.20)*
**BASC - Anxiety**		
Birth (Cord)	2.57 (−1.73, 6.88)	-0.06 (-2.99, 2.88)
1 Year	0.99 (−1.25, 3.23)	0.66 (−0.97, 2.29)
2 Year	1.05 (−1.27, 3.37)	0.66 (−1.05, 2.36)
3 Year	1.11 (−1.28, 3.49)	0.64 (−1.14, 2.42)
4 Year	1.15 (−1.30, 3.59)	0.62 (−1.23, 2.47)
5 Year	1.18 (−1.30, 3.66)	0.59 (−1.31, 2.48)
8 Year	1.16 (−1.30, 3.62)	0.42 (−1.52, 2.36)
12 Year	0.73 (−1.24, 2.71)	0.10 (−1.58, 1.78)
**CDI-II Total**		
Birth (Cord)	0.67 (−2.26, 3.60)	
1 Year	0.67 (−1.14, 2.48)	
2 Year	0.77 (−1.09, 2.62)	
3 Year	0.87 (−1.02, 2.75)	
4 Year	0.96 (-0.94, 2.86)	
5 Year	1.05 (-0.85, 2.96)	
8 Year	1.24 (-0.58, 3.05)	
12 Year	0.95 (-0.55, 2.44)	
**SCARED Total**		
1 Year	2.00 (-2.49, 6.48)	
1 Year	-0.43 (-3.06, 2.20)	
2 Year	-0.36 (-3.09, 2.38)	
3 Year	-0.27 (-3.11, 2.56)	
4 Year	-0.17 (-3.09, 2.75)	
5 Year	-0.06 (-3.04, 2.93)	
8 Year	0.31 (-2.72, 3.33)	
12 Year	-0.15 (-2.62, 2.32)	

[a] Adjusted for: A principal component analysis for caregiver anxiety and depression, maternal education (high school graduate or less, college graduate or above), marital status (married, unmarried), child age (continuous, years), child sex (male, female), caregiver relationship (continuous) and race (Non-Hispanic Black, White and Other; including White, non-Hispanic; Hispanic; American Indian; Asian/Pacific Islander; and Other).
*Marginally significant at p≤.10
**Statistically significant at p≤.05

© 2026 Hoover C et al. *JAMA Network Open*.

eFigure 1. Directed Acyclic Graph of exposures, outcomes, and covariates.

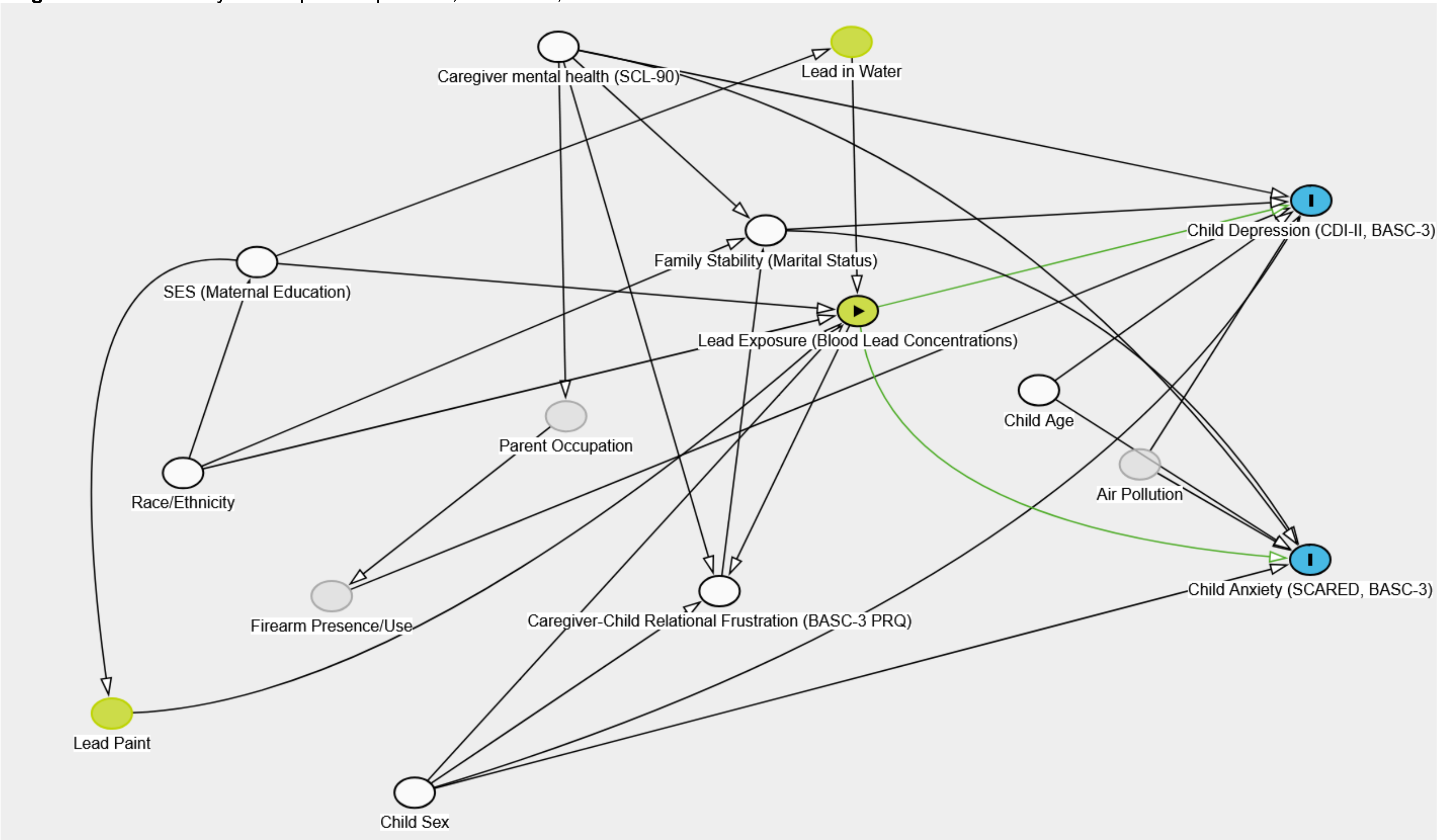

Blue are outcomes, green with triangle are exposures, green are ancestors of exposures, white are covariates conditioned upon, pink are confounders.

© 2026 Hoover C et al. *JAMA Network Open*.

eFigure 2. Adjusted difference in child, caregiver, and combined (cluster size of 2) BASC-3 outcomes per standard deviation increase in $\log_2$-transformed average childhood blood lead concentrations (rescaled by SD of full sample): The HOME Study (n=218).[a]

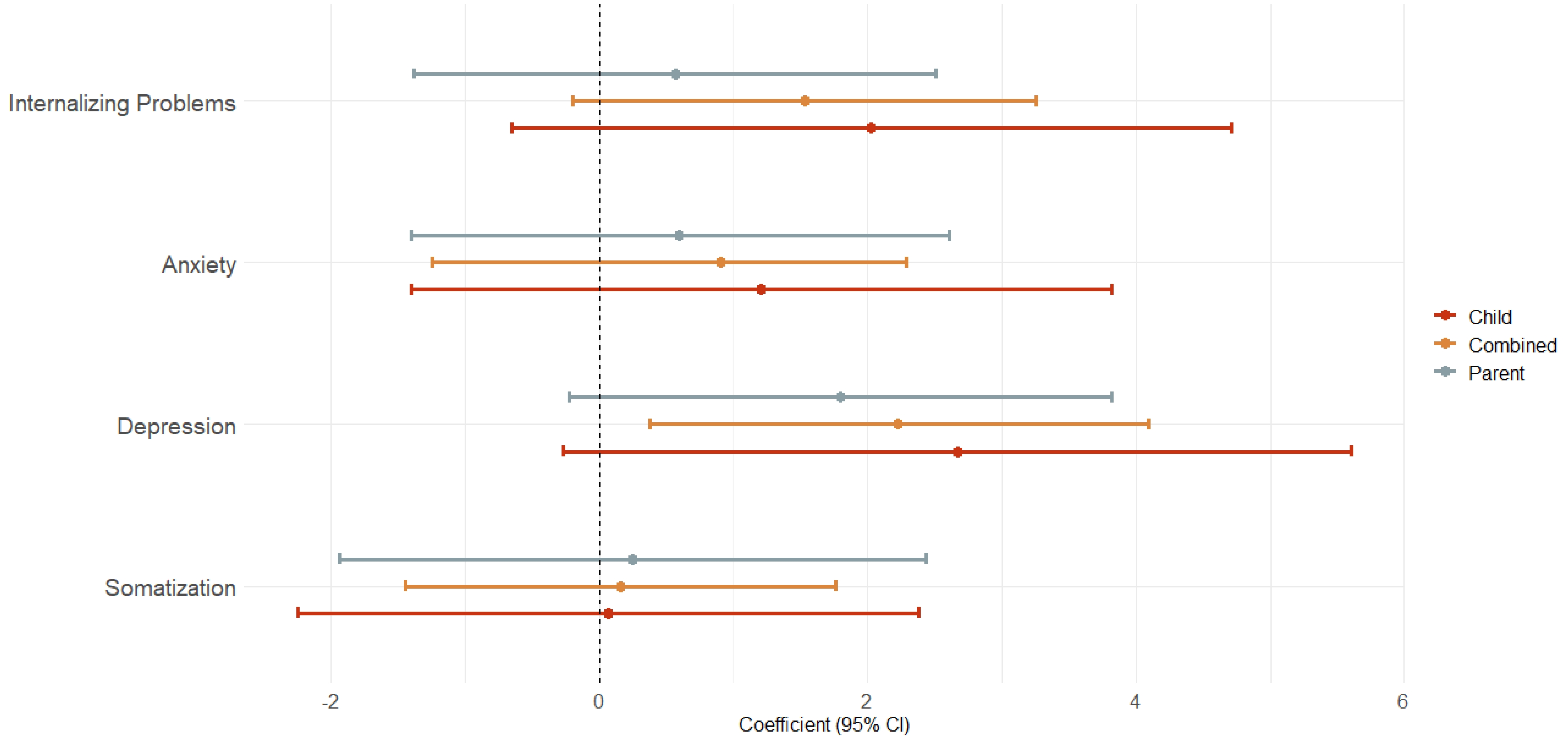

Combined results derived from linear regression models with GEE (compound symmetric covariance matrix) to account for the correlation of repeated measures within participants (i.e., mother- and child-report), ensuring robust standard errors and valid inference despite the potential non-independence of observations.

[a] Adjusted for: A principal component analysis for caregiver anxiety and depression, maternal education (high school graduate or less, college graduate or above), marital status (married, unmarried), child age (continuous, years), child sex (male, female), caregiver relationship (continuous) and race (Non-Hispanic Black, White and Other; including White, non-Hispanic; Hispanic; American Indian; Asian/Pacific Islander; and Other).

© 2026 Hoover C et al. *JAMA Network Open*.

eFigure 3. Unadjusted and adjusted[a] difference in CDI-II (depression) scores per standard deviation change in the log_2-transformed average childhood blood lead concentrations (n=217) (rescaled by standard deviation of full study sample): The HOME Study (n=218).

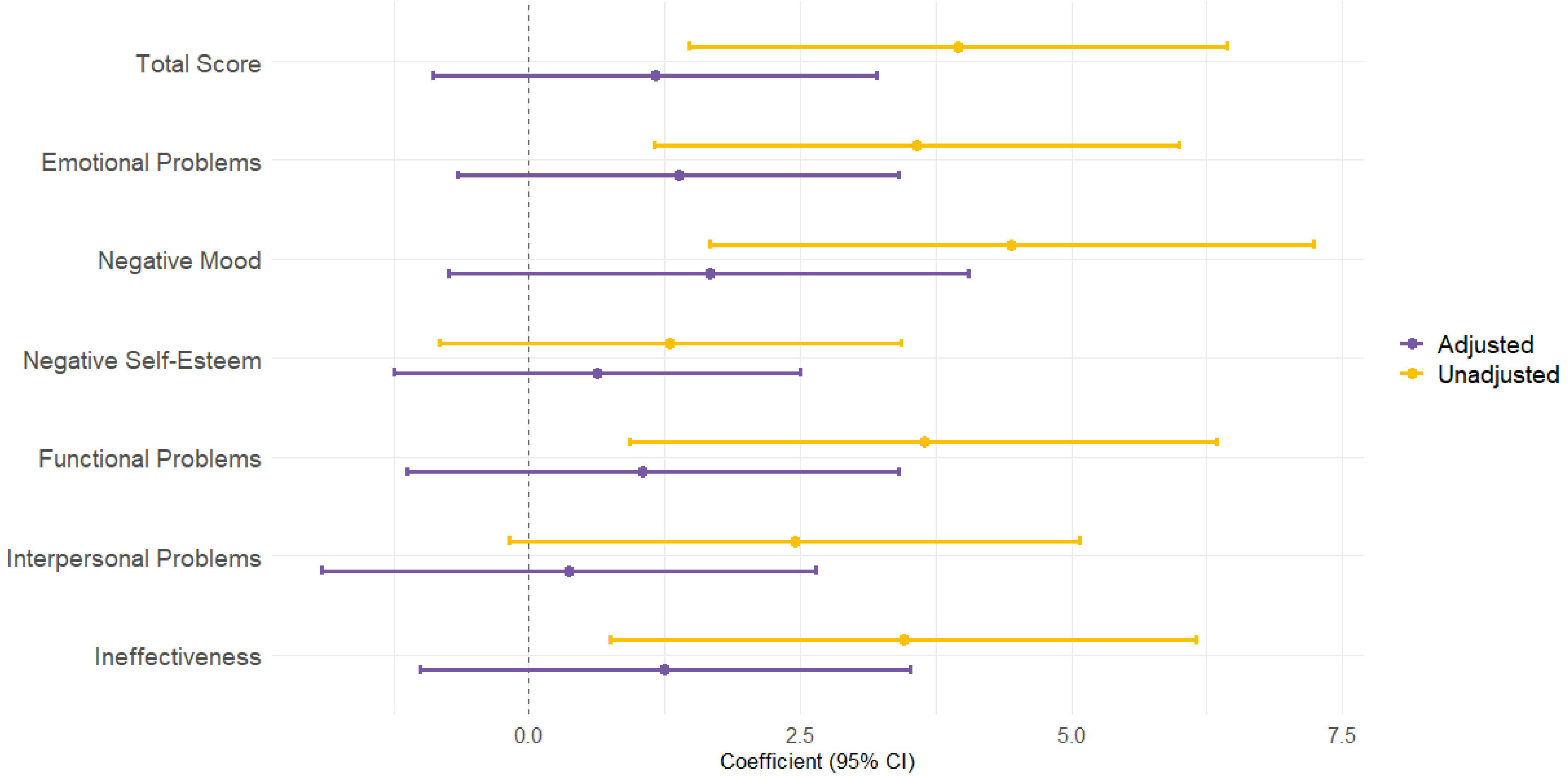

Results derived from linear regression models.

[a] Adjusted for: A principal component analysis for caregiver anxiety and depression, maternal education (high school graduate or less, college graduate or above), marital status (married, unmarried), child age (continuous, years), child sex (male, female), caregiver relationship (continuous) and race (Non-Hispanic Black, White and Other; including White, non-Hispanic; Hispanic; American Indian; Asian/Pacific Islander; and Other).

[b] Depressive symptoms measured with Child Depression Inventory (CDI)

© 2026 Hoover C et al. *JAMA Network Open*.

eFigure 4. Unadjusted and adjusted[a] differences in SCARED (anxiety) scores per standard deviation change in the log-transformed average childhood blood lead concentrations (rescaled by standard deviation of full study sample): The HOME Study (n=218).

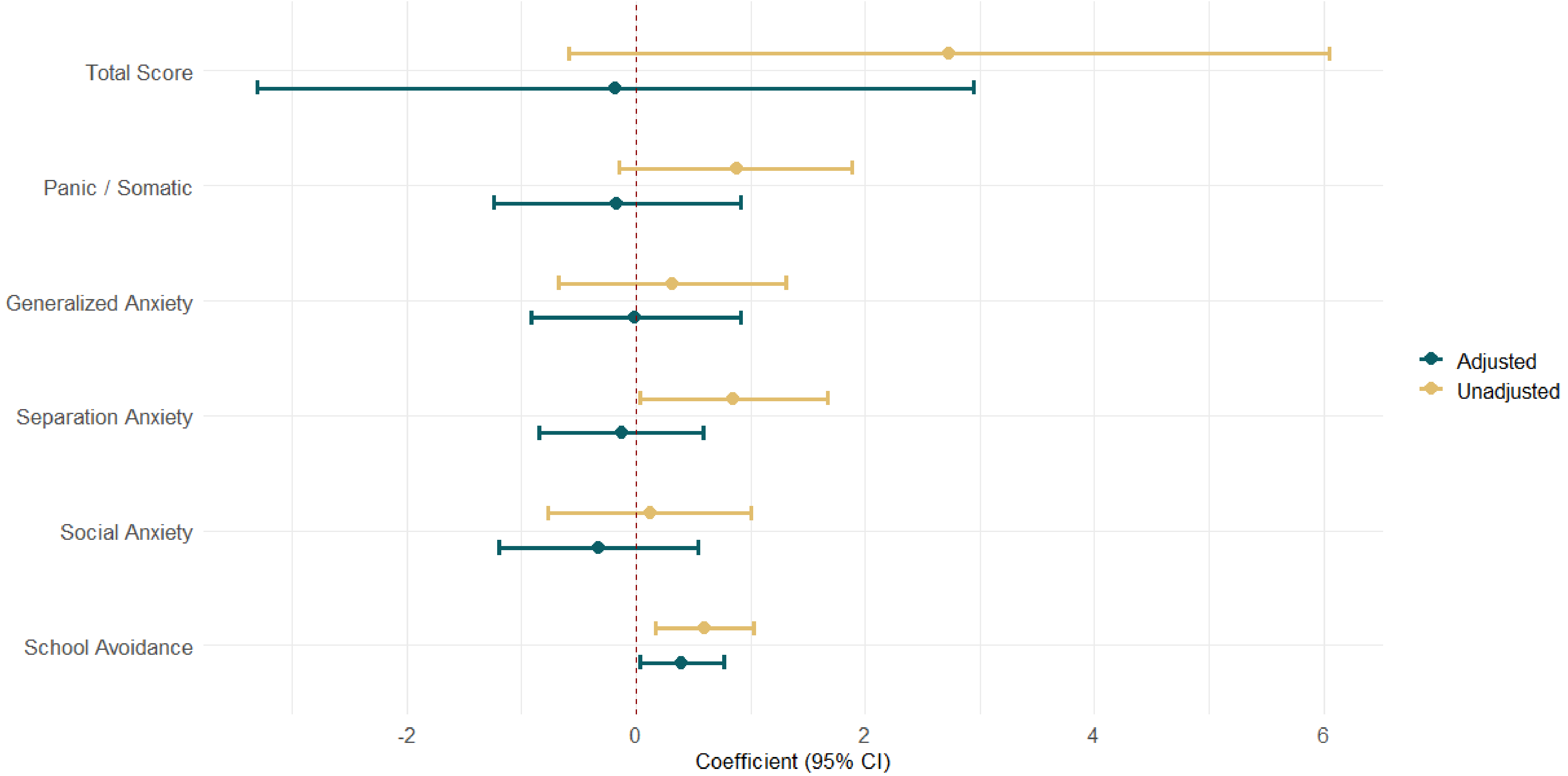

[a] Adjusted for: A principal component analysis for caregiver anxiety and depression, maternal education (high school graduate or less, college graduate or above), marital status (married, unmarried), child age (continuous, years), child sex (male, female), caregiver relationship (continuous) and race (Non-Hispanic Black, White and Other; including White, non-Hispanic; Hispanic; American Indian; Asian/Pacific Islander; and Other).
[b] Anxiety symptoms measured with Screen for Child Anxiety Related Disorders (SCARED)

© 2026 Hoover C et al. *JAMA Network Open*.

eFigure 5. Serial whole blood lead concentrations (μg/L) across childhood (n=339): The HOME Study.

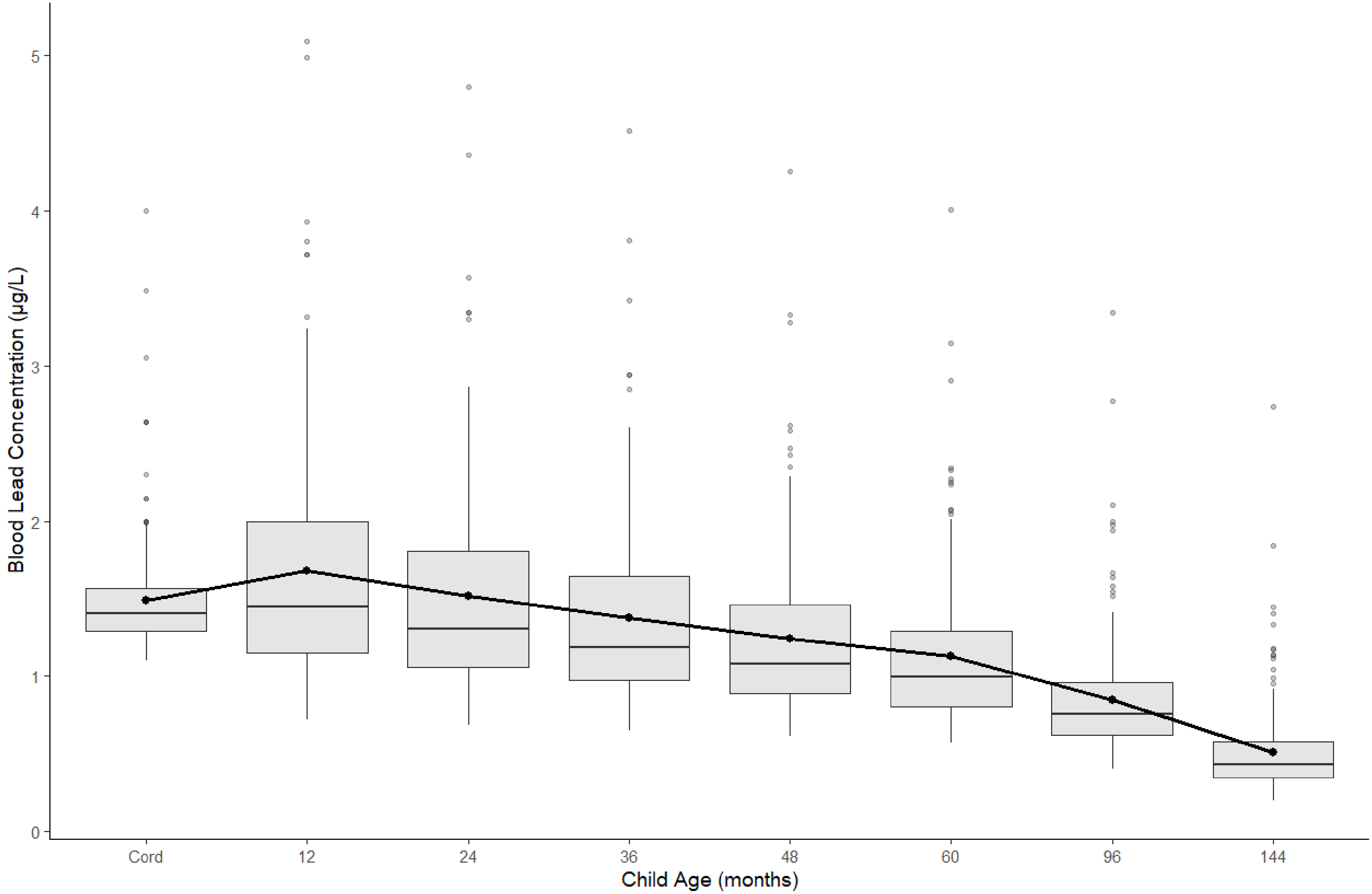

Boxes represent the interquartile range (25th–75th percentile), horizontal lines represent median values, and whiskers represent values within 1.5× the interquartile range. Circles denote outliers. Solid black points and connecting lines indicate mean blood lead concentrations at each time point.

© 2026 Hoover C et al. *JAMA Network Open*.
