# Supplementary material for: Blood Lead Concentrations and Depressive and Anxiety Symptoms in Childhood
Source: JAMA Netw Open. 2026 Jan 28;9(1):e2556019. doi: 10.1001/jamanetworkopen.2025.56019 (PMC12853203; doi:10.1001/jamanetworkopen.2025.56019)
Supplement: Supplement 2. — Data Sharing Statement [file jamanetwopen-e2556019-s002.pdf]

Data Sharing Statement

Hoover. Blood Lead Concentrations and Depressive and Anxiety Symptoms in Childhood. *JAMA Netw Open*. Published online January 28, 2026. doi:10.1001/jamanetworkopen.2025.56019

Data
Data available: No
